# Supplementary material for: Predictors of Response to Hydroxyurea and Switch to Ruxolitinib in HU-Resistant Polycythaemia VERA Patients: A Real-World PV-NET Study
Source: Cancers (Basel). 2023 Jul 21;15(14):3706. doi: 10.3390/cancers15143706 (PMC10377857; doi:10.3390/cancers15143706)
Supplement: Supplementary file 1 [file cancers-15-03706-s001.zip › cancers-2490898-supplementary.pdf]

Supplementary Table S1. Main patients' characteristics at diagnosis in patients with partial/no response to hydroxyurea (HU) who continued HU (HU-POOR) and who switched to ruxolitinib (HU-RUX).

**Characteristics**	**HU-POOR (n. 283)**	**HU-RUX (n. 114)**	***p* value**
Age, median (range), years<br>Age ≥ 65 years	66 (22-89)<br>161 (56.9%)	62 (21-79)<br>45 (39.5%)	<0.001<br><0.001
Male sex, no. (%)	150 (53%)	70 (61.4%)	0.13
JAK2^{V617F} VAF ≥ 50%, no. (%) on 251 evaluable	101/193 (52.3%)	34/58 (58.6%)	0.40
Platelet count, median (range), x 10^9/L	436 (138-1070)	474 (143-1209)	0.21
Leukocytes, median (range), x 10^9/L	10 (1-27.3)	10.4 (4.2-25.6)	0.28
Hemoglobin, median (range), g/dL<br>Male<br>Female	 18.6 (12-23.4)<br>17.5 (13.2-21.9)	 18.6 (13.4-23)<br>17.5 (13.8-20.5)	 0.36<br>0.84
Haematocrit, median (range), %<br>Male<br>Female	 56 (40.5-73)<br>54.4 (42.6-72)	 56.8 (38-70)<br>54 (39-65.8)	 0.71<br>0.97
Palpable spleen, no. (%) of 383 evaluable	97/277 (35%)	54/106 (50.9%)	0.004
Pruritus, no. (%)	95 (33.6%)	65 (57%)	<0.001
BMI ≥ 25, % of 284 evaluable	102/213 (47.9%)	42/71 (59.2%)	0.10
Charlson comorbidity index (CCI) ≥ 1, no. (%)	114 (40.3%)	46 (40.4%)	0.99
At least one CVRF, no. (%)	240 (80.8%)	85 (68.6%)	0.002
Thromboses pre-/at diagnosis, no. (%)	72 (25.4%)	30 (26.3%)	0.86
Antiplatelet agents, no (%)	223 (78.8%)	84 (73.7%)	0.14

Supplementary Table S2. Causes of death in patients with complete response to hydroxyurea (HU-CR) and in patients with partial/no response to hydroxyurea who continued HU (HU-POOR) and who switched to ruxolitinib (HU-RUX).

	PV Cohort (n.563)		HU-CR (n.166)		HU-POOR (n.283)		HU-RUX (n.114)	
**Causes of death**	**n.**	**Incidence rate (per 100 patient-years)**	**n.**	**Incidence rate (per 100 patient-years)**	**n.**	**Incidence rate (per 100 patient-years)**	**n.**	**Incidence rate (per 100 patient-years)**
**Blast Phase**	7	0.26	4	0.53	3	0.25	0	0
**Myelofibrosis**	4	0.15	2	0.27	2	0.17	0	0
**Infection**	5	0.15	1	0.13	3	0.25	1	0.14
**Second neoplasia**	9	0.34	5	0.66	4	0.33	0	0
**Thrombosis**	1	0.04	0	0	1	0.08	0	0
**Other cause**	10	0.38	3	0.40	7	0.59	0	0
